# Arterial bleeding during endoscopic ultrasound-guided pancreatic pseudocyst drainage using a novel ultrasound processor

**DOI:** 10.1055/a-2779-5162

**Published:** 2026-01-30

**Authors:** Junya Sato, Kazunari Nakahara, Yosuke Igarashi, Yusuke Satta, Akihiro Sekine, Yu Matsuda, Keisuke Tateishi

**Affiliations:** 1 Department of Gastroenterology, St. Marianna University School of Medicine, Kawasaki, Japan

Bleeding during endoscopic ultrasound-guided pancreatic pseudocyst drainage (EUS-PPD) is most often associated with electrocautery puncture or tract dilation 1 2 3 4 5 . Conversely, bleeding caused solely by fine-needle puncture is rare. We report a case of arterial bleeding induced by fine-needle puncture performed using a novel ultrasound processor ( Video 1 ).

Arterial bleeding induced by fine-needle puncture performed using a novel ultrasound processor, followed by endoscopic hemostasis.Video 1

**Fig. 1:**
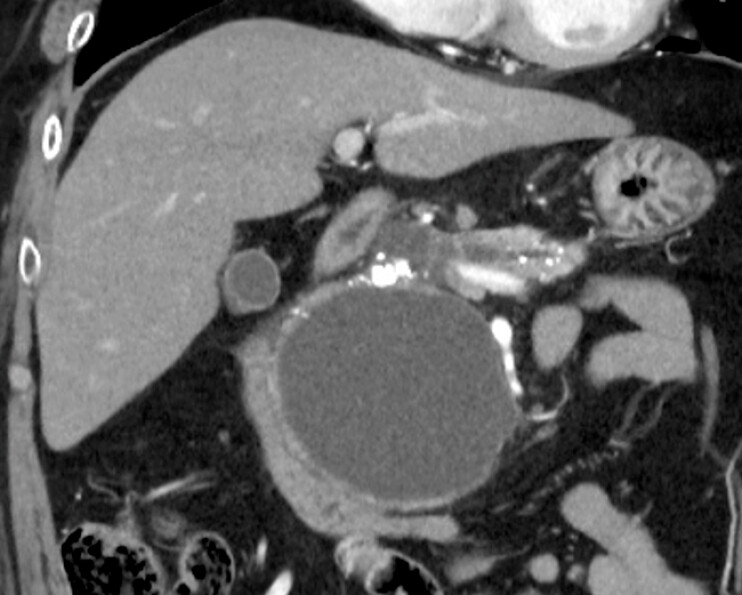
Computed tomography showing a 9-cm pseudocyst in the pancreatic head.

**Fig. 2:**
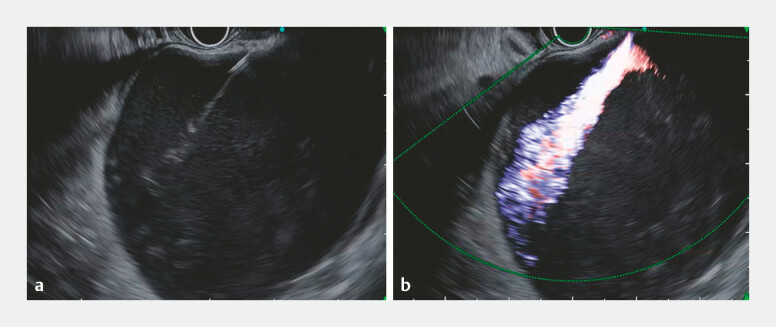
**a** Gray-scale ultrasound showing marked arterial spurting from the puncture site into the cyst cavity immediately after the withdrawal of the needle. **b** Color Doppler imaging confirming the presence of pulsatile arterial flow.

**Fig. 3:**
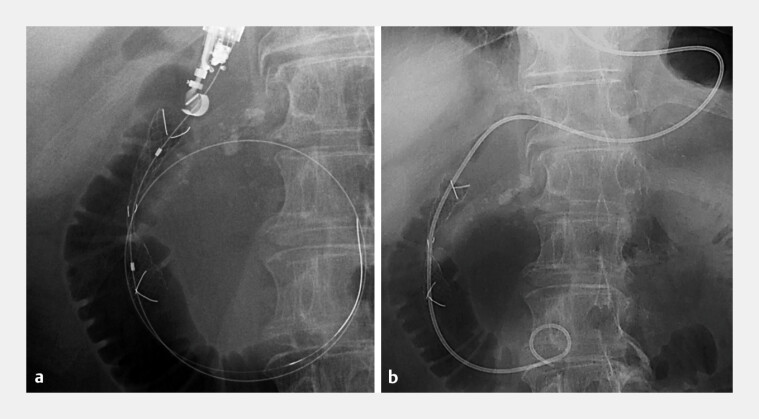
**a** Placement of a fully covered self-expandable metal stent deployed across the bleeding site resulting in complete hemostasis. **b** A nasal catheter inserted through the stent.

**Fig. 4:**
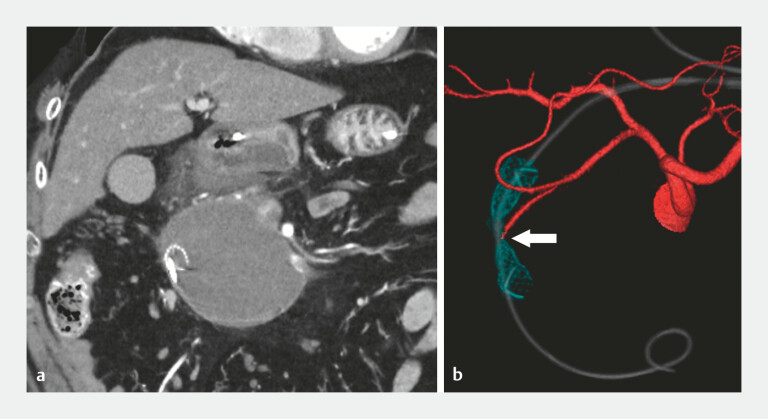
**a** Postprocedural computed tomography (CT) showing no extravasation. **b** The reconstructed CT image suggesting injury to the posterior superior pancreaticoduodenal artery (arrow).

A 61-year-old man with a 9-cm pseudocyst in the pancreatic head underwent EUS-PPD ( Fig. 1 ). After Doppler evaluation confirmed no intervening vessels, the pseudocyst was punctured from the duodenum with a 22-gauge needle (EZ Shot 3 Plus, Olympus) under the guidance of a novel ultrasound processor (EU-ME3, Olympus, Japan) and an ultrasound endoscope (GF-UCT260, Olympus). We inserted a 0.018-inch guidewire and subsequently removed the needle. We then immediately observed marked arterial spurting into the cyst cavity on gray-scale imaging ( Fig. 2 **a** ). Color Doppler confirmed pulsatile flow from the puncture site into the cyst cavity ( Fig. 2 **b** ). Despite inserting a 7-Fr dilator (ES Dilator, Zeon Medical Co., Japan) for compression hemostasis, bleeding recurred upon its withdrawal. Therefore, a 10-mm fully covered self-expandable metal stent (FCSEMS; HILZO biliary stent, ABIS Inc., Japan) was deployed across the EUS-guided created route, resulting in complete hemostasis. Then, a nasal catheter was placed through the FCSEMS ( Fig. 3 ). Postprocedural computed tomography showed no extravasation; however, injury to the posterior superior pancreaticoduodenal artery was suspected ( Fig. 4 ). The patient experienced no further bleeding, and 1 month later, the FCSEMS was removed without complications.

**Fig. 5:**
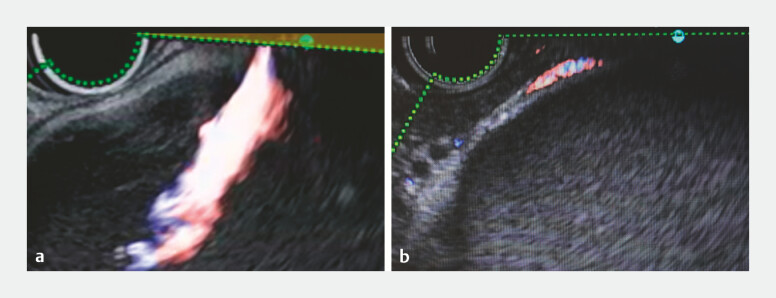
Color Doppler imaging comparison between the ( **a** ) EU-ME3 and ( **b** ) EU-ME2 ultrasound processors. The EU-ME3 processor displays a slightly reduced color Doppler imaging area (yellow highlight) near the gastrointestinal wall compared with the EU-ME2.

This case highlights the possibility of bleeding during EUS-PPD even when Doppler imaging reveals no visible vessels and a thin needle is used. Compared with its predecessor (EU-ME2 model), the EU-ME3 processor detected certain small areas near the gastrointestinal wall lacking blood-flow signals ( Fig. 5 ). Thus, caution must be exercised to avoid inadvertent vessel injury near the gastrointestinal wall.

Endoscopy_UCTN_Code_CPL_1AL_2AD
